# Outcomes of ECLS-SHOCK Eligibility Criteria Applied to a Real-World Cohort

**DOI:** 10.3390/jcm12226988

**Published:** 2023-11-08

**Authors:** Dirk von Lewinski, Lukas Herold, Eva Bachl, Heiko Bugger, Theresa Glantschnig, Ewald Kolesnik, Nicolas Verheyen, Martin Benedikt, Markus Wallner, Friederike von Lewinski, Albrecht Schmidt, Stefan Harb, Klemens Ablasser, Michael Sacherer, Daniel Scherr, Martin Manninger-Wünscher, Sascha Pätzold, Johannes Gollmer, Andreas Zirlik, Gabor G. Toth

**Affiliations:** 1 Department of Internal Medicine, Division of Cardiology, Medical University of Graz, Auenbruggerplatz 15, 8036 Graz, Austria; eva.bachl@stud.medunigraz.at (E.B.); heiko.bugger@medunigraz.at (H.B.); ewald.kolesnik@medunigraz.at (E.K.); nicolas.verheyen@medunigraz.at (N.V.); markus.wallner@medunigraz.at (M.W.); albrecht.schmidt@medunigraz.at (A.S.); stefan.harb@medunigraz.at (S.H.); michael.sacherer@medunigraz.at (M.S.); daniel.scherr@medunigraz.at (D.S.); martin.manninger-wuenscher@medunigraz.at (M.M.-W.); sascha.paetzold@medunigraz.at (S.P.); johannes.gollmer@medunigraz.at (J.G.); gabor.g.toth@medunigraz.at (G.G.T.); 2 Department of Internal Medicine, Division of Endocrinology and Diabetology, Medical University of Graz, Auenbruggerplatz 15, 8036 Graz, Austria; 3 Interdisciplinary Metabolic Medicine Trials Unit, Medical University of Graz, 8036 Graz, Austria

**Keywords:** cardiogenic shock, myocardial infarction, mechanical support, VA-ECMO, mortality, lactate

## Abstract

Background: Cardiogenic shock (CS) exhibits high (~50%) in-hospital mortality. The recently published Extracorporeal life Support in Cardiogenic Shock (ECLS-SHOCK) trial demonstrated the neutral effects of the use of veno-arterial extracorporeal membrane oxygenation (VA-ECMO) on all-cause death, as well as on all secondary outcomes in subjects presenting with myocardial-infarction (MI)-related CS. Here, we compared ECLS-SHOCK eligibility criteria with a real-world cohort of CS patients. Methods and Results: ECLS-SHOCK eligibility criteria were applied to a prospective single-center CS registry (the PREPARE CS registry) consisting of 557 patients who were consecutively admitted to the catheterization laboratory (cath lab) of the Medical University of Graz, Austria, due to CS (SCAI C-E). Overall use of mechanical circulatory support (MCS) in this cohort was 19%. Sixty-nine percent of the entire cohort had MI-related CS, 38% of whom would have met ECLS-SHOCK eligibility criteria, thus representing only 27% of the PREPARE CS registry. Exclusion from the ECLS-SHOCK trial was based on patients with initial lactate values below 3 mmol/L (n = 168; 43.6%), aged over 80 years (n = 65; 16.9%), and with a duration of cardiopulmonary resuscitation (CPR) exceeding 45 min (n = 22; 5.7%). The 30-day mortality of patients of the PREPARE CS registry who met the ECLS-SHOCK eligibility criteria was 57.0%, compared to 48.4% of patients in the ECLS-SHOCK trial. The patients’ baseline characteristics, however, differed considerably with respect to type of infarction, age, and gender. Conclusions: In a real-world cohort of patients with MI-related CS, only 38% of patients met the eligibility criteria of the ECLS-SHOCK trial. Thus, the impact of the use of VA-ECMO on outcome parameters in MI-related CS, as observed in the ECLS-SHOCK trial, may differ in a more heterogeneous real-world CS population of the PREPARE CS registry.

## 1. Introduction

Acute myocardial infarction (AMI) is complicated by cardiogenic shock (CS) in up to 10% of cases [1] and represents the most frequent single cause of the development of CS. However, in recent studies, other conditions, such as acute decompensated chronic heart failure, have an increasing share in causing CS t [2]. Despite advances in acute and intensive care, early mortality from CS remains high [3,4]. In fact, a ST-elevation myocardial infarction (STEMI) registry reported that 76% of 7-day mortality of STEMI patients was due to cardiogenic shock [5]. With respect to mechanical support, the intra-aortic balloon pump (IABP) was the first and one of the most-used devices because it was easy to implant through a relatively small access. However, it predominantly played a role in decreasing afterload, as increasing the effects on cardiac output are limited. In recent decades, veno-arterial-extracorporeal membrane oxygenation (VA-ECMO) was considered as the last-resort emergency treatment option for refractory CS, to ensure organ perfusion until recovery of cardiac function, escalation to assist long-term device treatment, or as bridging in cases of cardiac transplantation [6]. Among other treatment options, VA-ECMO is suggested in the position statements of the European Society of Cardiology Association of Acute Cardiovascular Care (ACVC) [7] and the American Heart Association (AHA) [8].

While various decision algorithms have been published to facilitate the selection of patients who might benefit from VA-ECMO [9], hardly any data of adequately powered, prospective, and randomized trials was available [10,11,12].

Importantly, the very recently published randomized Extracorporeal life Support in Cardiogenic Shock (ECLS-SHOCK) trial provided neutral 30-day follow-up results on the primary endpoint—all-cause mortality—as well as on all secondary endpoints, including renal replacement therapy, repeat revascularization, and myocardial infarction, as well as poor neurologic outcomes or lengths of hospital stay for VA-ECMO use in individuals with MI-related CS. The primary endpoint was also neutral in predefined subgroups of age, sex, or diabetic status, as well as for subgroups based on their type or localization of myocardial infarction, their need for cardiopulmonary resuscitation, or the level of baseline lactate with a threshold of 6 mmol/L. Moreover, these neutral efficacy data were accompanied by inferior safety outcomes in the VA-ECMO group, driven by increased rates of peripheral ischemic vascular complications, as well as by increased rates of moderate or severe bleeding. Unsurprisingly, this pattern was confirmed in a recent meta-analysis of four randomized trials, including a total of 567 patients, of which 420 patients originated from the ECLS-SHOCK trial [13]. Nevertheless, this initiated a debate about the usefulness of mechanical circulatory support (MCS) for such patients [14].

However, considering the wide spectrum of clinical phases of CS, it remains unclear whether these neutral trial results can be applied to a real-world CS cohort. We hypothesized that patients included in the ECLS-SHOCK trial represent only a minor portion of total MI-related CS patients in a real-world setting. Moreover, baseline characteristics and outcomes may differ between patients that do or do not fulfil ECLS-SHOCK eligibility criteria.

## 2. Methods

PREPARE CS [9] is a prospective single center registry, including all patients who underwent cardiac catheterization due to CS at the University Heart Center Graz, Austria, within a consecutive 4-year period from May 2019 to May 2023. At this center, approximately 1200 acute-coronary-syndrome patients are treated per year. In this study, CS was defined as a hemodynamically compromised condition requiring vasopressor support, matching the Society for Cardiovascular Angiography and Interventions (SCAI) classifications C to E [9]. During the observation period, 557 patients were included in the registry. Of these 557 patients, 385 patients suffered from MI-related CS. The MCS devices used in the PREPARE CS registry were the Impella CP^®^ (Abiomed), Aachen, Germany pump and the Xenios ECMO platform (Xenios, Heilbronn, Germany). The intra-aortic ballon pump (IABP) device was not used in patients within this registry. All devices were implanted by the interventional cardiologist, via femoral access. In the VA-ECMO cases, the arterial cannula was connected to an antegrade arterial sheath (6F) to ensure adequate perfusion of the leg. The choice of MCS was mainly based on the assessment of residual circulation, as well as on ventilation capacity. Patients with a residual, moderate-to-severe circulatory reserve qualified primarily for Impella CP^®^, while patients with severe circulatory reserve to no circulatory reserve qualified for VA-ECMO. Patients with marked oxygenation insufficiency, expressed by the Horowitz index, also qualified for VA-ECMO. The present analysis compared clinical characteristics and 30-day mortality of MI-related CS patients from the PREPARE CS registry with those from the ECLS-SHOCK trial. MI-related CS patients included in the PREPARE CS registry were characterized according to whether they would have qualified for the ECLS-SHOCK trial (PREPARE CS ELIGIBLE) or not (PREPARE CS NON-ELIGIBLE). 

The study was approved by the Ethics Committee of the Medical University of Graz, Austria (EK 31–323 ex 18/19), and was conducted in full conformity with the 1964 Declaration of Helsinki and all subsequent revisions, as well as in accordance with the guidelines of the International Conference on Harmonization for Good Clinical Practice (ICH GCP E6 guidelines).

## 3. Statistics

Data are presented as mean (±standard deviation) or median [interquartile range], as appropriate. For the PREPARE CS data, normal distribution was tested using the Pearson omnibus normality test. Continuous variables were compared by the unpaired Student’s *t*-test or the Mann–Whitney test, as appropriate. Categorical variables were compared with Fisher’s exact or chi-square test, as appropriate, and the results are expressed in an odds ratio (OR) with a 95% confidence interval (CI). A value of *p* < 0.05 was considered as significant. These data were compared to the published data of the ECLS-SHOCK trial.

## 4. Results

### 4.1. Patient Characteristics

Baseline characteristics, as well as comorbidities (arterial hypertension, diabetes mellitus, hyperlipoproteinemia), were comparable between the PREPARE CS ELIGIBLE patients and the ECLS-SHOCK patients. Key baseline parameters of cardiogenic shock, such as lactate levels (5.8 [4.4; 8.5] vs. 6.9 [4.6; 9.9] mmol/L, respectively), pH (7.23 [7.12; 7.30] vs. 7.2 [7.1; 7.3], respectively), systolic blood pressure (95 [78; 114] vs. 96 [80; 120] mmHg, respectively), and the proportion of resuscitated patients (76.4% vs. 77.7%, respectively; *p* = 0.73) were also comparable. However, the median age was higher in the PREPARE CS ELIGIBLE patients, differing by two years from the ECLS-SHOCK patients (65 [58; 73] vs. 63 [56; 70], respectively), and significant differences were also observed for sex (28.4% vs. 18.7% female, respectively; *p* = 0.01) and the subgroups of MI with higher rates of STEMI in the PREPARE CS ELIGIBLE patients than in the ECLS-SHOCK patients (79.7% vs. 66.2%, respectively; *p* < 0.01; Table 1). In contrast to these two groups, the PREPARE CS NON-ELIGIBLE cohort was characterized by even higher median ages (71 years [60; 80]), lower lactate levels (2 mmol/L [1.3; 2.7]), and higher blood pH (7.36 [7.25; 7.40]), respectively, representing an older but less severely shocked population. The rate of cardiopulmonary resuscitation (CPR) before admission was also significantly lower (50%) than in either the PREPARE CS ELIGIBLE cohort (76%, *p* < 0.01) or the ECLS-SHOCK trial (78%, *p* < 0.01). 

Overall use of MCS in the PREPARE CS registry was 20%, but considerably higher in the PREPARE CS ELIGIBLE subgroup (30%). Of these 30%, representing 44 patients, 20 were treated with VA-ECMO, 19 were treated with Impella, and five were treated with the combination of both (ECMELLA) (Table 1).

### 4.2. Outcomes

Based on initial values for lactate, pH, systolic blood pressure, and the need of CPR, patients meeting ECLS-SHOCK eligibility criteria (i.e., ECLS-SHOCK patients and the PREPARE CS ELIGIBLE group) were more severely ill than those in the PREPARE NON-ELIGIBLE group or the overall PREPARE CS cohort. Therefore, outcome data were primarily compared between the groups that fulfilled ECLS-SHOCK criteria. Compared to the ECLS-SHOCK cohort, 30-day mortality was higher (56.8 vs. 48.4%) in PREPARE CS ELIGIBLE patients, whereas time in the intensive care unit (ICU) (5 [2; 12] vs. 9 [4; 15] days), total length of hospital stay (8 [2; 20] vs. 11 [4; 19] days), and days on a respirator (3 [1; 7] vs. 6 [4; 10] days) were considerably shorter in this group (Table 2). With respect to safety outcomes, the most frequent were BARC 3–5 bleedings, both in PREPARE CS patients and ECLS-SHOCK patients. The bleeding rate was comparable between PREPARE CS MI patients (10%) and the control group of ECLS-SHOCK (9.6%) patients, where more than a quarter of the patients still received MCS therapy (Impella, IABP, or VA-ECMO) as a bail-out strategy, as allowed by the trial protocol. The nmbers of stroke and peripheral complications were lower in the PREPARE CS ELIGIBLE group compared to the ECLS-SHOCK group, but their definition was less precise than it was in the trial.

The outcomes in the PREPARE NON-ELIGIBLE group were better, despite the fact that first-day mortality was not different to that of those register patients who would have been eligible for the ECLS-SHOCK trial (13% vs. 14%). The lower intrahospital mortality rate of 41% was accompanied by the shortest durations of ICU stay (3 [2; 8] days) and time on respirator (0 [0; 5] days), despite unchanged total length of hospital stay (8 [4; 15] days). With respect to safety outcomes, BARC 3–5 bleeding was only seen in 6% of these patients. 

## 5. Discussion

This analysis revealed significant differences in baseline characteristics between the ECLS-SHOCK trial cohort and a real-world registry, including consecutive cardiogenic shock patients referred to a cath lab of a large tertiary center. Considerable differences remained even if the ECLS-SHOCK eligibility criteria were applied to the registry cohort.

In the absence of timely medical actions, the hemodynamics and the clinical condition of patients with CS rapidly deteriorated and worsened patients’ prognosis. MCS devices are considered as last-resort support for these patients. However, the identification of patients who may benefit from MCS implantation remained unclear and was mainly based on registry or single-center data, due to a lack of prospective randomized trials. 

Early data on VA-ECMO use in MI patients with severe CS indicated beneficial effects. However, the analysis was based on historical data covering an observation period of 16 years and on comparing outcomes before and after the availability of VA-ECMO. Since interventional and pharmacological therapies for AMI considerably improved over the observation period, interpreting these data in the context of contemporary clinical practice is challenging [15]. More recent data are available from the ECMO-SHOCK trial, including analysis of a large proportion of MI patients with immediate VA-ECMO use vs. primarily conservative treatment, but excluding the large group of comatose cardiac-arrest survivors [12]. Throughout the trial, a significant outcome differences were not detected for the composite endpoint of death from any cause, resuscitated circulatory arrest, or the implementation of another MCS at 30 days, nor for prespecified secondary endpoints, including all-cause mortality at 30 days, neurological outcomes and safety endpoints such as clinically significant bleeding, leg ischemia, pneumonia, sepsis, and technical complications between the treatment arms. However, more than a third of the patients in the conservative group crossed over and received MCS. Assessment of the true effect of VA-ECMO was further complicated by the fact that, despite considerable technological advances and improvements in cardiovascular patient management, VA-ECMO therapy is still accompanied by a high rate of safety issues, such as bleeding, vascular complications, and infection, which impact the net clinical outcomes [16].

The ECLS-SHOCK trial aimed to clarify whether VA-ECMO implantation may be beneficial for 30-day mortality or secondary endpoints in individuals suffering MI-related cardiogenic shock. Overall, the trial demonstrated neutral effects for all efficacy outcomes, while revealing an increased hazard for ischemic vascular complications and bleeding. When interpreting safety data, however, one has to bear in mind that the occurrence of vascular complications and bleeding increase with the duration of VA-ECMO treatment. Thus, the longer a patient survives on VA-ECMO, the higher the rate of safety issues. As in previous MCS trials, treatment groups of ECLS-SHOCK were characterized by considerable numbers of patients who could not be treated as initially planned. In ECLS-SHOCK, 8.1% of patients who were intended to be treated with VA-ECMO did not receive the device, while 12.5% of patients in the group that was not intended to be treated with VA-ECMO had such a device implanted within 24 h. In addition, another 15.4% of the patients in the control group were treated using other mechanical circulatory support (27 by Impella and one by IABP). The use of MCS in the ECLS-SHOCK control group was, therefore, almost identical with the the use of MCS in the PREPARE CS ECLS ELIGIBLE subgroup (see Table 2) and still exceeded the frequency of use of MCS in the total registry cohort, as well as the current use of MCS for cardiogenic shock patients in Germany [16]. Although the crossover rates in the ECLS-SHOCK trial did not exceed those of previous trials, they still hindered the interpretation of the data. As detailed individual data are not yet available, it can only be speculated whether these crossover patients reduced the mortality rate in the control group, as this bail-out strategy might have improved 30-day survival. Hence, the bail-out use of MCS in refractory cardiogenic shock might, potentially, represent an important indication of VA-ECMO or other MCS use. On the other hand, very early use in patients deteriorating during a catheter intervention might also represent a subgroup of patients who might benefit from MCS use, but they can hardly be included in prospective trials by the typically unplanned and very acute setting of these scenarios. With this in mind, and considering the lack of alternatives, it is of utmost importance for clinical practices to interpret these results in the context of a real-world CS population. 

In our real-world registry, only 148 of the 385 patients (38%) with MI-related cardiogenic shock would have qualified for the ECLS-SHOCK trial. Non-eligible patients were predominantly in rather early phases of CS, as indicated by lactate levels below 3 mmol/L (44%), while a more-advanced condition of CS in a minority of patients was indicated by prolonged CPR (6%). Hence, optimal treatment, as well as the role of MCS in these groups, remain unclear. Beyond that, even real-world eligible patients (i.e., PREPARE CS ELIGIBLE group) showed relevant differences in terms of clinical characteristics, such as age and sex, compared to the ECLS-SHOCK population. The relevant literature, as well as PREPARE CS data, suggest that at least a quarter of CS patients are female [17,18]; this includes prospective trials such as the ECMO-CS trial, with 26.5% female patients. Women, however, remained markedly underrepresented in ECLS-SHOCK, comprising less than 19% of the entire cohort. Moreover, while octogenarians were excluded in ECLS-SHOCK, they represent at least one-sixth of all CS patients in various registries and analyses [8,19]. This group is known to have a particularly bad prognosis in cardiogenic shock. Finally, clinical presentation also deviates from real-word data, with a larger portion of NSTEMI cases in the trial. Whether these differences would have affected the overall outcome of the ECLS-SHOCK trial is speculative. 

In any event, the use of VA-ECMO requires considerable human, structural, and financial resources [16]. For these reasons, it should be allocated to patients in an appropriate and resource-efficient manner. Identifying pre-ECMO predictors of in-hospital survival is crucial to achieving this goal. 

The neutral results of the ECLS-SHOCK trial might impact clinical use and reimbursement for MCS in the future, which could be observed after the IABP-SHOCK trial, with a consecutive decline of IABP use. However, due to the poor prognosis of patients with cardiogenic shock, IABP was quickly replaced by other forms of MCS [20]. A recent analysis calculated the average hospital costs to be EUR 40.000 in MCS-treated MI-related cardiogenic shock patients in Germany [16]. Considering the hemodynamic support of VA-ECMO, there is currently no proper alternative that could provide full replacement of native circulation. Axial pumps, such as the Impella device, primarily unload the left ventricle, provide only partial hemodynamic support, and cannot directly impact on oxygenation and decarboxylation. The ongoing prospective, multicenter, open-label Danish-German cardiogenic shock trial for patients with MI-related cardiogenic shock assesses the effects of Impella CP in these patients. The inclusion criteria are broad, but patients who are comatose after out-of-hospital cardiac arrest are excluded. In this trial, the timing of MCS implantation is recommended to be prior to angioplasty. The primary endpoint will be all-cause mortality at 180 days. The results are eagerly awaited, and they might provide sufficient data to fill this gap in the evidence [21]. Moreover, the timing of MCS initiation in MI-related cardiogenic shock remains unclear. The ECLS-SHOCK trial reported a balanced insertion rate of VA-ECMO, before and during revascularization. Data on differences between these two approaches are, however, not yet available. Considering all of these factors, the ECLS-SHOCK results should probably be seen as the greatest motivation for research and device development, in order to find solutions for the weaknesses in the current technology. As well as leading to a better understanding of the pathophysiological mechanisms of CS, future research may help to properly identify patients who will benefit from MCS.

## 6. Limitations

This analysis was limited by its nature—i.e., comparing registry data with a randomized trial. Nevertheless, our comparison sheds new light on the trial’s data set and allows the date to be considered in the context of real-world practices.

## 7. Conclusions

While the ECLS-SHOCK trial demonstrated no benefit of VA-ECMO in the treatment of MI-related CS, this analysis implied that the real-world CS population may expand far beyond ECLS-SHOCK eligibility criteria. Therefore, the use of MCS in CS patients who present in the cath lab in the early or late phases of the CS cascade may require individualized decision-making.

## Figures and Tables

**Table 1:** Baseline characteristics.

	PREPARE CS All n = 557	PREPARE CS MI n = 385	PREPARE CS—ECLS-SHOCK ELIGIBLE n = 148	PREPARE CS–ECLS- SHOCK NON-ELIGIBLE n = 237	ECLS-SHOCK n = 417
age, median (IQR 1; 3)	68 (59; 78)	68 (58; 78)	65 (58; 73)	71 (60; 80)	63 (56; 70)
female gender, n (%)	159 (29)	110 (29)	42 (28)	68 (29)	78 (19)
known diabetes, n (%)	143 (26)	102 (27)	46 (31)	56 (24)	130 (31)
known hypertension, n (%)	315 (57)	214 (56)	76 (51)	138 (58)	233 (56)
known hyperlipidemia, n (%)	190 (34)	133 (30)	37 (25)	96 (41)	129 (31)
CPR IHCA OHCA	358 (64) 138 (25) 220 (40)	231 (60) 110 (29) 121 (31)	113 (76) 43 (29) 70 (47)	118 (50) 67 (28) 51 (22)	324 (78)
intubated at arrival	357 (64)	221 (57)	118 (80)	103 (44)	No data available
MI, n (%) STEMI NSTEMI	385 (69) 289 (52) 96 (17)	385 (100) 289 (75) 96 (25)	148 (100) 118 (80) 30 (20)	237 (100) 171 (72) 66 (28)	417 (100) 276 (66) 141 (34)
lactate at admission [mmol/L], median (IQR 1; 3)	3.6 (1.7; 6.5)	3.3 (1.7; 6.0)	5.8 (4.4; 8.5)	2 (1.3; 2.7)	6.9 (4.6; 9.9)
pH at admission, median (IQR 1; 3)	7.28 (7.16; 7.37)	7.30 (7.18; 7.38)	7.23 (7.12; 7.30)	7.36 (7.25; 7.40)	7.2 (7.1; 7.3)
systolic BP at admission [mmHg], median (IQR 1; 3)	100 (83; 117)	99 (83; 116)	95 (78; 114)	100 (85; 118)	96 (80; 120)
MCS, n (%) VA ECMO Impella ECMELLA	105 (19) 42 (8) 50 (9) 13 (2)	76 (20) 29 (8) 36 (9) 11 (3)	44 (30) 20 (14) 19 (13) 5 (3)	32 (14) 9 (4) 17 (7) 6 (3)	246 (59) 218 (52) 28 (7) 17 (4)

Myocardial infarction (STEMI/NSTEMI), RRSyst < 90 mmHg or catecholamines. Exclusion criteria: CPR > 45 min, mechanical cause of CS, ECLS non-poss due to pAVK, age < 18 or >80 years.

**Table 2:** Outcomes.

	Prepare CS All n = 557	PREPARE CS MI n = 385	PREPARE CS ELIGIBLE * n = 148	PREPARE CS NON-ELIGIBLE n = 237	ECLS-SHOCK n = 417
intrahosp/30 d mortality, n (%)	280 (50)	182 (47)	84 (57)	98 (41)	202 (49)
first day mortality, n (%)	82 (15)	51 (13)	21 (14)	30 (13)	no data available
length of ICU stay [days], median (iqr 1; 3)	4 (2; 10)	4 (2; 10)	5 (2; 12)	3 (2; 8)	9 (4; 15)
length of hospital stay [days], median (iqr 1; 3)	8 (2; 18)	8 (3; 17)	8 (2; 20)	8 (4; 15)	11 (4; 19)
respirator [days], median (iqr 1; 3)	2 (0; 6)	1 (0; 6)	3 (1; 7)	0 (0; 5)	6 (4; 10)
overall complications, n (%) bleeding stroke * vascular *	74 (13) 68 (12) 3/198 (2) 13/198 (7)	39 (10) 38 (10) 3/131 (2) 10/131 (8)	24 (16) 22 (15) 1/51 (2) 4/51 (8)	15 (6) 15 (6) 2/80 (3) 6/80 (8)	114 (27) 69 (17) 14 (3) 31 (7)

* Stroke and vascular complications were not recorded over the whole period of the registry.

## Data Availability

The data presented in this study are available on request from the corresponding author. The data are not publicly available due to privacy restrictions.

## Abbreviations

ACVC	Association for Acute Cardiovascular Care of the European Society of Cardiology
AHA	American Heart Association
AMI	acute myocardial infarction
bp	blood pressure
CI	confidence interval
CPR	cardiopulmonary resuscitation
CS	cardiogenic shock
ECLS	extracorporeal life support
IABP	Intra-aortic balloon pump
ICU	intensive care unit
IHCA	in-hospital cardiac arrest
MCS	mechanical circulatory support
MI	myocardial infarction
NSTEMI	non-ST-segment elevation myocardial infarction
OHCA	out-of-hospital cardiac arrest
OR	odds ratio
SCAI	Society for Cardiovascular Angiography and Interventions
STEMI	ST-segment elevation myocardial infarction
VA-ECMO	veno-arterial-extracorporeal membrane oxygenation
